# The South African Medicines Control Council: Comparison of Its Registration Process With Australia, Canada, Singapore, and Switzerland

**DOI:** 10.3389/fphar.2019.00228

**Published:** 2019-03-14

**Authors:** Andrea Keyter, Sam Salek, Shabir Banoo, Stuart Walker

**Affiliations:** ^1^Department of Clinical and Pharmaceutical Sciences, School of Life and Medical Sciences, University of Hertfordshire, Hatfield, United Kingdom; ^2^Department of Health, South African Health Products Regulatory Authority, Pretoria, South Africa; ^3^Department of Pharmacy and Pharmacology, University of the Witwatersrand, Johannesburg, South Africa; ^4^Centre for Innovation in Regulatory Science, London, United Kingdom

**Keywords:** South African regulatory review, Medicine Control Council (MCC), TGA, Health Canada, Swissmedic, HSA

## Abstract

**Introduction:** Comparisons between regulatory authorities of similar size and regulatory characteristics facilitate value-added benchmarking and provide insight into regulatory performance. Such comparisons highlight areas for improvement as authorities move toward achieving their regulatory goals and stakeholders’ demands. The aims of this study were to compare the registration process and the regulatory review model of the South African Medicines Control Council (MCC) to that of four other similar-sized regulatory authorities and to identify areas for improvement that may inform recommendations to the South African Health Products Regulatory Authority (SAHPRA) as it looks to re-engineer and enhance the registration process in South Africa.

**Methods:** A questionnaire describing the organisational structure, the registration process, good review and decision-making practices of the MCC was completed by the author (AK) for the purpose of this study and validated by the Registrar of the MCC. Similar questionnaires were also completed and validated by Australia’s Therapeutic Goods Administration (TGA), Canada’s Health Canada, Singapore’s Health Science Authority (HSA) and Switzerland’s Swissmedic.

**Results:** A comparison of the MCC regulatory process with the four comparative agencies indicated that they all have similar requirements and employ a full-review model although the timelines for the MCC were considerably longer. However, similar quality measures were implemented by all authorities as part of their good review practices (GRevP) including prioritising transparency, communication, continuous improvement initiatives and training.

**Conclusion:** Comparisons made through this study provided insight into the areas of the MCC registration process that may be improved and have informed recommendations to SAHPRA including the implementation of facilitated regulatory pathways, definition of targets for key milestones in regulatory review and formal implementation and monitoring of GRevP. In order to build quality into the review process the application of a standardised template for the clinical assessment of medicines such as the Universal Methodology for Benefit-Risk Assessment (UMBRA) could be considered as well as enhancing transparency and communication through the application of an electronic management system and the development of publicly available summaries for the basis of approval.

## Introduction

Efforts toward regulatory harmonisation and convergence have been evident over the last 20 years which has been supported through the initiation of both regulatory authorities and the pharmaceutical industry. The impact of these efforts has translated into globally standardised technical regulations and requirements for the quality, efficacy, and safety of medicines and their improved access by patients (WHO, 2000). While each country has autonomy in the manner in which it effects its regulatory mandate in line with national requirements, it is recognised that there is value in benchmarking regulatory models and sharing best practices (Mashaki Ceyhan et al., 2018). Comparisons between regulatory authorities of similar size, regulatory mandates, structures, resource characteristics and regulatory challenges would be more beneficial than comparisons between authorities with vastly different characteristics and competencies (Mashaki Ceyhan et al., 2018). Regulatory authorities in jurisdictions within the emerging pharmaceutical markets would benefit from comparisons with other mature regulatory authorities of similar size such as Health Canada and the Australia’s Therapeutic Goods Administration (TGA; Hashan et al., 2016).

The national regulatory authority (NRA) of South Africa is mandated through the Medicines and Related Substances Act, 1965 (Act 101 of 1965) to ensure the efficient, effective and ethical assessment and registration of medicines and medical devices that meet defined standards of quality, safety, efficacy and performance and to ensure that the process of assessing and registering medicines and medical devices is transparent, fair, objective and concluded within an appropriate time frame (Republic of South Africa, 2017). The drive for the establishment of a more effective regulatory framework in South Africa has been evident over the past two decades. In June 2017, the Medicine and Related Substances Act, 1965 (Act 101 of 1965), was amended to allow for the transition of the NRA of South Africa, formerly known as the Medicines Control Council (MCC) to the South African Health Products Regulatory Authority (SAHPRA). The transition from MCC to SAHPRA has been described in full by Keyter et al. (2018).

This new era provided an opportunity to study the regulatory processes applied by the MCC with a view to enhancing the regulatory review process and the responsiveness of the NRA as it moves toward effecting its improved regulatory landscape as SAHPRA. As SAHPRA moves forward with its objective for regulatory reform, it is important that the authority has the relevant capabilities and decision-making frameworks in place to ensure the efficient application of resources with a view to improving overall approval times and patients’ access to new medicines (Keyter et al., 2018). The former regulatory performance of the MCC should serve as a baseline from which SAHPRA may monitor progress and achievements whilst benchmarking planned reform against that of other NRAs in order to identify the strengths and areas for improvement.

A comparative study of the regulatory performance of the MCC registration process with that of other regulatory authorities in the developed and emerging markets has not been previously performed. Therefore, there is a need for such a study as the South African NRA strives to become a reference NRA in the African region. Similar studies have been performed to compare the Turkish Medicines and Medical Devices Agency (Mashaki Ceyhan et al., 2018), the Saudi Food and Drug Authority (Hashan et al., 2016) and the Jordan Food and Drug Administration (Haqaish et al., 2017) with the regulatory authorities of Australia, Canada and Singapore. This study aimed to compare the registration process of the MCC in South Africa with the processes of Australia, Canada, Singapore and Switzerland in order to identify the strengths, challenges and areas of improvement within the regulatory review processes applied by the MCC and to assess the level of implementation of quality measures, good review practices (GRevPs), decision-making principles and continuous improvement initiatives within the MCC operations, as it strives toward the goals of improved regulatory performance under the auspices of SAHPRA.

## Materials and Methods

### Study Participants

This study provides a comparison of the registration process historically administered by South Africa’s NRA, until recently known as the MCC, against that of four other NRAs. The four regulatory authorities selected for this study were based on the size of the agencies and the patient population they served, the year since established and the nature of the review model (full assessment) applied. The data for the comparator agencies was collected in 2014 and subsequently updated in 2017. Thus, the five regulatory authorities included in this study were as follows: the Therapeutic Goods Administration (TGA) of Australia, Health Canada, the Health Sciences Authority (HSA) of Singapore, Swissmedic and the MCC of South Africa.

### Study Tool and Data Collection Process

The questionnaire used in the study was completed by the author (AK) and validated by the then Registrar of the MCC in 2017 to describe the regulatory review system for market authorisation of new active substances (NASs) as applied by the MCC and the overall review time of NASs from the date of application to the date of approval during the period 2015–2017 (Keyter et al., 2018). The questionnaire (McAuslane et al., 2009), developed to facilitate capturing data pertaining to regulatory systems in emerging market jurisdictions with respect to their implementation of GRevP, was used in this study. Data were collected using a standardised format to allow for appropriate comparison and analyses of information collected from multiple regulatory authorities. The questionnaire consists of four parts: part 1 – structure of the regulatory authority, the resources available and the review models applied by the authority; part 2 – regulatory review process using a standardised process map format to allow for ease of comparison; part 3 – indicators and description of the measures that have been implemented to build quality into the regulatory review process and decision-making practices and the implementation of GRevP to ensure transparent, consistency and timely regulatory review outcomes; and part 4 – identifies the enablers and barriers to quality decision making. The completion of the questionnaire and preparation of the report by the author were validated by the Registrar of the MCC. Similar questionnaires were completed by the Head of the licensing (registration) division of TGA, Health Canada, the HAS, and Swissmedic and the validated country reports that were prepared to describe the regulatory systems applied in each of these countries were used to inform the results of this study. The questionnaire used in this study was designed to allow for simple comparative analyses of the structure, processes, and practices of international regulatory authorities (McAuslane et al., 2009; Mashaki Ceyhan et al., 2018).

### Models of Regulatory Review

Regulatory authorities may apply different regulatory pathways requiring stratified levels of data assessment depending on the type of medicine under review and the regulatory status of the medicine in other reference or benchmark jurisdictions. There are three types of product review assessments used by regulatory authorities: the verification review (Type I); abridged (Type II); and full review (Type III) (McAuslane et al., 2009).

#### Review Assessment Type I – Verification Model

This model avoids duplication of regulatory effort through the recognition of the regulatory decisions made by one or more reference or benchmark authorities (McAuslane et al., 2009). This model is built on the premise that the regulatory authority has verified the data submitted for compliance with the reference country(s) authorisation(s).

#### Review Assessment Type II – Abridged Model

The abridged model makes provision for a truncated review focused on the evaluation of clinical data (benefit-risk assessment) as well as country specific requirements related to quality of the product (e.g., stability) (McAuslane et al., 2009). In this model it is a prerequisite that the product has been approved for market authorisation by a reference or benchmark regulatory authority.

#### Review Assessment Type III – Full Review Model

The full review model is intended for use by regulatory authorities that have the necessary resources to perform a full independent scientific review of NASs applications (McAuslane et al., 2009). The full review model does not require evidence of marketing authorisation from any other regulatory authority at the time of submission.

### Ethics Committee Approval

The study protocol received approval from the University of Hertfordshire Institutional Ethics Committee. Since the study participants were regulatory authority staff, it did not require the local Ethics Committee approval.

## Results

### Comparative Assessment of Regulatory Review Processes and Milestones

**FIGURE 1:**
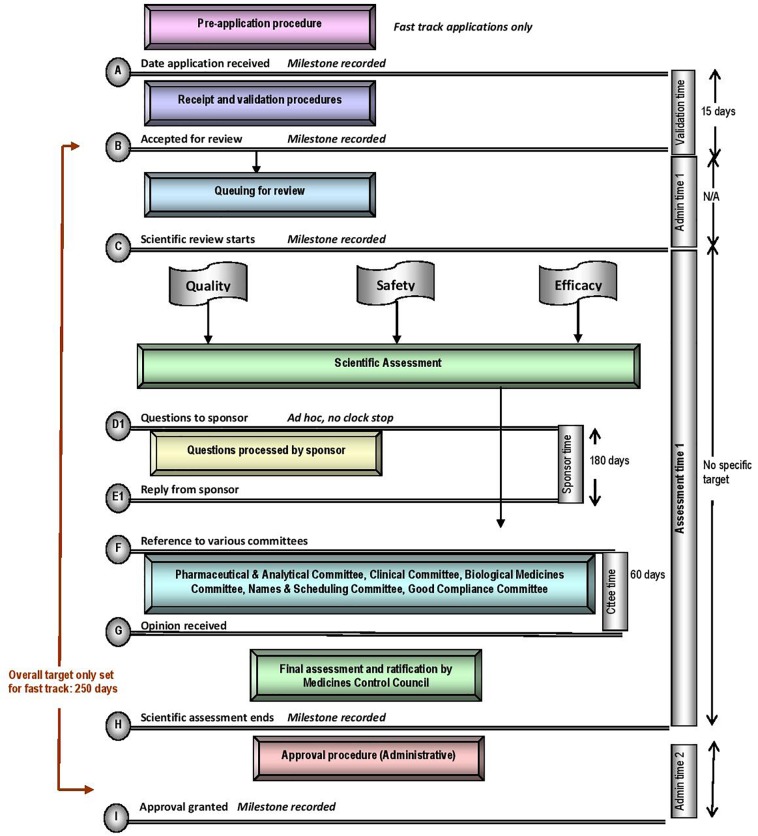
Registration process map for South Africa, calendar days.

The five regulatory authorities compared in this study have similar mandates for regulating medicines for human use. They are responsible for ensuring that harmonised standards for market authorisation of such products are applied whilst ensuring timely access to medicines that are safe, effective and of good quality. Regulatory authorities have demonstrated autonomy in the manner in which they execute their mandates, however, differences may be observed within their regulatory review processes, timelines and the application of GRevPs. The regulatory review processes applied by the MCC is depicted in the standardised process map (Figure 1) (Keyter et al., 2018). The map provides a simple representation of the review and authorisation of applications for NASs and MLEs that are approved on the first cycle, but does not include generic medicines, biosimilars, complementary medicines, veterinary medicines or medical devices. The map does not describe the process, in the event that the application was refused.

The MCC conducted a Type III full assessment in the review of all applications, including NASs, major line extensions (MLEs) and generics for orthodox, biological, complementary, and veterinary medicinal products. A full independent assessment of quality, efficacy and safety data is performed and an application for market authorisation for NASs and MLEs may be submitted to the MCC prior to approval by any other regulatory authority worldwide. The MCC did not place any reliance on or consider the review performed by any other NRAs. The TGA, Health Canada, HSA, and Swissmedic also perform Type III full assessments and a Certificate of Pharmaceutical Product (CPP) is not required at the time of submission (Table 1). A Type II abridged review is employed by the TGA if requested by the sponsor and if the product has been approved by one or more reference authorities, by Swissmedic but only for selected applications, mainly generic medicine applications and by the HSA only if the product has been approved by one or more authorities. The HSA also conducts Type I verification review but only if the product has been approved by two or more authorities. While Health Canada are planning to implement this reliance pathway, Swissmedic intends to roll this out by 2019 (Health Canada, 2018).

**Table 1:** Models of assessment of the five agencies and extent of the scientific review.

Type of review model	South Africa (MCC)	Australia	Canada	Switzerland	Singapore
Verification review (type I)	x	x	x	x	✓^a^
Abridged review (type II)	x	✓^b^	x	✓^c^	✓^d^
Full review (type III)	✓	✓	✓	✓^e^	✓
**EXTENT OF SCIENTIFIC REVIEW**					
1. Chemistry, manufacturing, and control (CMC) data					
Extensive assessment	✓	✓	✓	✓	✓
2. Nonclinical data					
Extensive assessment	✓	✓	✓	✓	✓^f^
3. Clinical data					
Extensive assessment	✓	✓	✓	✓	
**ADDITIONAL INFORMATION OBTAINED (WHERE APPROPRIATE)**					
Other agencies’ internal review reports	✓	✓	✓	✓	x
				*(Occasionally)*
Reports on the internet	✓	✓	✓	✓	✓
				*(Occasionally)*
General internet search	✓	✓	✓	✓	✓
				*(Occasionally)*


*^a^Only if the product has been approved by two or more reference agencies. ^*b*^Only if requested by the sponsor and if the product has been approved by two or more reference agencies. ^*c*^Used for selected applications, mainly generic applications. ^*d*^Only if the product has been approved by one or more reference agencies. ^*e*^Used mainly for applications for NAS. ^*f*^Only for biological and biosimilar products.*

### Data Requirements

The MCC and HSA do not have a formal pre-application procedure in place, however, Swissmedic offers this in cases of a priority review, while, for Type III full reviews, the HSA may require the sponsor to submit a notification of intent to apply for market authorisation. The TGA and Health Canada have formalised this process and consider it as an opportunity to familiarise reviewers with the product, potentially uncover any major areas of concern early in the registration process, identify the potential for priority review and provide a platform for the sponsor to discuss their submission and obtain scientific advice.

The MCC required the full chemistry, manufacturing and control (CMC) data, nonclinical data and clinical data to be submitted in the Common Technical Document (CTD) format to support the application for market authorisation. The other four comparative regulatory authorities also request full CMC, nonclinical and clinical datasets and also conduct an extensive assessment of these datasets for a Type III full review. All five of the regulatory authorities perform a review of quality, safety and efficacy data in parallel and pricing negotiations are separate from the technical review of the data submitted. The primary scientific review of the data is performed by internal technical staff of the four comparative regulatory authorities, with the possibility of seeking advice from contracted external experts on an *ad hoc* basis. The quality assessment of NASs and MLEs conducted by the MCC was performed by both internal technical staff and external reviewers while the assessment of clinical data for NASs and MLEs was reviewed by external reviewers only.

Committee structures within the four comparative NRAs are similar in that the NRAs engage with various expert committees on an *ad hoc* basis to support the scientific review process and to provide scientific advice and expert opinion on selected dossiers. The committee structure within the MCC was different in that all assessment reports would be channelled to various scientific committees for expert opinion and the final regulatory decision would be taken by the Medicines Control Council.

All five regulatory authorities are members of the Pharmaceutical Inspection Co-operation Scheme (PIC/S) (PIC/S, 2018) and have implemented processes to ensure that evidence of the good manufacturing practice (GMP) status of the manufacturer is provided during the review process. Sponsors may submit a copy of the GMP certificate issued by a reference agency as evidence of a manufacturers GMP status, however, if the GMP status of the manufacturer cannot be confirmed at the time of application for market authorisation, the regulatory authority may conduct a GMP inspection at the manufacturing site in parallel to the review process.

### Target and Approval Times

The MCC review process consisted of application receipt and validation procedures, queue time for allocation of applications to reviewers, a scientific review of CMC, nonclinical and clinical data conducted in parallel, company response and final authorisation through the regulatory decision taken by the Council. The milestone timelines for the MCC review procedures are displayed in Figure 1. A “fast track” status was assigned to eligible applications in order to expedite the registration of essential medicines. While the review process was the same for “fast track” applications, these applications would be prioritised over existing applications, queued for allocation to reviewers. The target set for the overall review time of fast track applications was 250 calendar days. The median approval times for fast track NAS applications approved in 2015, 2016, and 2017 were 1218, 921, and 609 calendar days, respectively (Keyter et al., 2018). There was no target time set for the overall review time of NASs, but the median approval times for NAS marketing authorisation applications approved in 2015, 2016, and 2017 were 1175, 1641, and 1466 calendar days, respectively (Keyter et al., 2018). These data demonstrated that the MCC was neither able to achieve the target timelines set for fast track applications nor meet the targets in 2015, 2016, and 2017 for the key milestones within the regulatory review process (Figure 1). The data presented by Keyter et al. (2018) represents the overall approval time based on the date of application and the date of registration; data that were routinely monitored and measured for the period 2015–2017. The median overall approval time does not include or account for sponsor response time and the time taken to reach the other milestones identified within the regulatory review process.

In comparison the TGA, Health Canada, the HSA and Swissmedic have set overall target review times, for standard full approvals, at 305 calendar days, 355 calendar days, 270 working days and 330 calendar days, respectively. The overall target review times set by these four regulatory authorities do include sponsor response time, unlike those for MCC. During the period 2013–2017, the TGA, Health Canada and Swissmedic achieved median approval times of 364, 350, and 487 days, respectively (Bujar et al., 2018). In 2017, Health Canada Swissmedic TGA approved 30, 29, and 24 NASs, respectively. Despite these numbers varying on an annual basis, the overall number of NASs approved by these authorities has increased (Bujar et al., 2018). The number of NAS approvals between 2008 and 2012 increased by 56% for TGA, 46% for Health Canada and 41% for Swissmedic when compared to the number of NASs approved between 2013 and 2017 (Bujar et al., 2018).

### Comparative Assessment of Good Review Practices

This study identified the quality measures that have been established and implemented by the five regulatory authorities with a view to comparing the aptitude and culture of the authorities in the application of these measures in order to ensure quality, transparency, consistency and continuous improvement in the regulatory review process.

#### Quality Measures

The quality measures evaluated in this comparative study are listed in Table 2. Swissmedic is the only regulatory authority in this comparative study that has a dedicated quality department and that has implemented all the listed quality measures. The MCC and TGA implemented six of the seven measures and Heath Canada and the HSA have implemented five quality measures. Only Health Canada and Swissmedic have formally implemented GRevPs while the other three authorities have informally implemented GRevPs. All of the five regulatory authorities occasionally participated in shared and joint reviews.

**Table 2:** The quality measures implemented by the five regulatory authorities.

Measure	Regulatory authority				
	South Africa (MCC) (6/7)	Australia (6/7)	Canada (6/7)	Switzerland (7/7)	Singapore (5/7)
Internal quality policy	✓	✓	✓	✓	x
	*(Informally)*
Good review practice system	✓	✓	✓	✓	✓
	*(Informally)*	*(Informally)*	*(Formally)*	*(Formally)*	*(Informally)*
Standard operating procedures for guidance of assessors	✓	✓	✓	✓	✓
	*(Informally)*
Assessment templates	✓	✓	✓	✓	✓
Dedicated quality department	x	x	x	✓	x
Scientific committee/s appointed	✓	✓	✓	✓	✓
Shared and joint reviews	✓	✓	✓	✓	✓
	*(Occasionally)*	*(Occasionally)*	*(Occasionally)*	*(Occasionally)*	*(Occasionally)*


*Informally: A policy/system/procedure is in place but is not formally documented. Formally: A policy/system/procedure is in place and is formally documented.*

#### Transparency and Communication

Improved transparency and communication are common goals for regulatory authorities worldwide. There are nine established transparency and communication parameters that may be implemented by regulatory authorities to enhance stakeholder relationships (Table 3). The MCC implemented seven out of the nine parameters. Currently the industry is unable to track the progress of applications. Although the MCC documented and communicated the summary of grounds for regulatory approval with the sponsor, this summary was not published or made available in the public domain. The HSA also does not publish the summary basis of approval or provide feedback to the industry on submitted dossiers. The TGA implemented all of the nine transparency and communication parameters while Swissmedic and Health Canada implemented eight and the HSA six of the nine measures (Table 3).

**Table 3:** Transparency and communication parameters in the five agencies.

Measure	Regulatory authority				
	South Africa (MCC) (7/9)	Australia (9/9)	Canada (8/9)	Switzerland (9/9)	Singapore (6/9)
Feedback to industry on submitted dossiers	✓	✓	✓	✓	x
Details of technical staff to contact	✓^a^	✓	✓	✓	✓
Pre-submission scientific advice to industry	✓^b^	✓	✓	✓	✓
Official guidelines to assist industry	✓	✓	✓	✓	✓
Industry can track progress of applications	x	✓	✓	✓	✓
Publication of summary of grounds on which approval was granted	x^c^	✓	✓	✓	x
Approval times	✓^d^	✓	✓	✓	✓
Advisory committee meeting dates	✓	✓	x	✓	x
Approval of products	✓	✓	✓	✓	✓


*^a^Contact details are made available on an ad hoc basis. ^*b*^Meetings are held with industry on an ad hoc basis. ^*c*^Summary is available but is currently not published. ^*d*^Approval times are not made available to the public.*

#### Continuous Improvement Initiatives

A comparison was made of the continuous improvement initiatives that have been implemented by the five regulatory authorities. Swissmedic implemented all five initiatives, the TGA and the HSA implemented four, Health Canada implemented three and the MCC implemented two of the five initiatives (Table 4). The MCC did not undergo routine external quality audits and did not perform routine internal quality audits. Further, reviews of assessors’ feedback were performed and the MCC carried out an informal review of feedback from stakeholders.

**Table 4:** Continuous improvement initiatives in the five regulatory authorities.

Measure	Regulatory authority				
	South Africa (MCC) (2/5)	Australia (4/5)	Canada (3/5)	Switzerland (5/5)	Singapore (4/5)
External quality audits	x	x	x	✓	x
Internal quality audits	x	✓	✓	✓	✓
Internal tracking systems	x	✓	✓	✓	✓
Reviews of assessors’ feedback	✓	✓	x	✓	✓
Reviews of stakeholders’ feedback	✓	✓	✓	✓	✓


#### Training and Education

Various types of training and education such as induction training, on-the-job training, attendance at internal and external courses, international workshops and secondments in other regulatory authorities can contribute to the development of personnel and the continuous improvement of the regulatory review process. All five of the regulatory authorities in this comparative study implemented all eight of the measures for training and education.

#### Enablers and Barriers to Good-Quality Decision Making

This study identified aspects that the MCC considered to be pivotal enablers in the effectiveness and efficiency of the MCC review process and decision-making procedures for NAS applications. These included the eagerness of the regulatory authority in South Africa to build confidence in the regulatory system, the minimal staff turnover at the MCC that contributed toward the retention of institutional knowledge and the support from scientific committees in the regulatory review of applications for market authorisation. The lack of an electronic document management system and outdated review processes coupled with fixed committee structures and decision-making processes were deemed to be barriers in effecting the regulatory mandate of the MCC in a timely manner. The other four authorities in the comparative study listed a variety of enablers that contributed to good decision making, with common themes of regulatory convergence and harmonisation and the implementation of GRevPs emerging as top enablers on the list. The barriers identified by these authorities included frustrations with incomplete submissions for market authorisation and the need for appropriate electronic systems to support the review process and allow for document management and a full integration of electronic business systems.

## Discussion

Regulatory authorities around the world strive to enhance their regulatory performance and in doing so ensure timely patients’ access to safe, good quality, effective medicines. A comparison of the regulatory systems and review processes implemented by NRAs globally contribute to the understanding of these challenges and inform solutions through sharing of best practices and lessons learned. The MCC recognised the importance of harmonisation and regulatory convergence and was striving to align itself with the systems and processes implemented by mature regulatory authorities in an effort to improve regulatory performance and ensure timely patient access to medicines. This study aimed to identify the similarities and differences between the registration processes applied by similar-sized mature regulatory authorities and those formerly applied by the MCC. This study demonstrated the strengths in the regulatory review process of the MCC and the areas that required improvement providing an evaluation of the regulatory performance of the MCC review model and reflected the progress by the MCC in applying GRevP. The TGA, Health Canada, the HSA and Swissmedic were selected for this study as authorities with similar regulatory characteristics and review models to allow for an appropriate comparison. In particular, these four agencies now have a work-sharing approach, which was another reason for this being an appropriate selection criteria of comparative agencies.

Over the past decade a number of regulatory authorities from the emerging markets have been evaluated using the questionnaire. Therefore, the four regulatory authorities selected as comparators for this study were based on the size of the agencies and the patient population they serve, the year since established and the nature of the review model (full review) applied. Furthermore, agencies from the emerging economies such as Tanzania and Kenya were not considered comparable to MCC because of the size of the agency and the population they serve, but in particular because South Africa carries out “full review” which is different to that of the other agencies in the region. It is also recognised that the FDA and the EMA are not appropriate agencies with which benchmark MCC. The reasons include both the size of the agency and the population they serve, but in particular the resources available (both in financial terms and the number of reviewers) to such agencies which in the case of the FDA include 1200 reviewers of whom 220 are statisticians. As regards EMA, being a consortium of 32 countries engaging rapporteur and co-rapporteur in the review process would constitute totally a different review model to that of South Africa.

### Review Type and Process

The MCC conducted a Type III full assessment for all NAS applications for market authorisation and such applications could be submitted to the MCC prior to approval by another NRA. In line with the other four comparative NRAs, the GMP status of the manufacturer is confirmed concurrently with the review process and a CPP is not required at the time of submission. The MCC participated in regional alignment initiatives and conducted shared or joint reviews with other regulatory authorities such as Zambia, Zimbabwe, Namibia, and Botswana (Regulatory Resources for Africa, 2015). However, no formal measures were put in place to ensure consistent quality during shared or joint reviews and participation in this initiative did not influence the way in which the MCC conducted reviews in general. A work-sharing programme is a creative way to maximise resources even when agencies are separated by time and distance. This was the rationale for the collaboration between the agencies in Australia, Canada, Switzerland and Singapore which now have established efficient work-sharing experience (McAuslane et al., 2017).

Considering the resource constraints that were faced by the MCC and the large volumes of applications for market authorisation received; it is beneficial to consider the use of facilitated registration pathways (FRPs) to expedite regulatory decisions and to enhance the re-engineered registration process envisaged by SAHPRA. Primary FRPs are defined as “pathways that speed the development, review and approval of a product; typically implemented by mature NRAs for a first nondependent review” (Liberti, 2016). Secondary FRP can be used to expedite regulatory decisions made by NRAs and contribute toward decreasing median approval times for medicines resulting in improved patient access to medicines. Secondary FRPs are based on the reliance or recognition of a prior review and regulatory decision made by another NRA. In this case, *reliance* is defined as the act whereby, in making a regulatory decision, an NRA in one jurisdiction considers, and in some cases, gives significant weight to the regulatory decision made by another NRA and *recognition* is defined as the routine acceptance of the regulatory decision made by another NRA (Liberti, 2016).

Applying FRPs that provide a risk-based approach for the review of applications for market authorisation may help to conserve limited resources and reduce regulatory burden by avoiding duplication of regulatory efforts (Alsager et al., 2015). This would be an advantage when considered in line with the recommendations of the WHO (Ward, 2014; WHO, 2014) by embracing regulatory harmonisation/convergence strategies; engaging in reliance and recognition activities that allow NRAs in resource-limited settings to consider or accept regulatory decisions made by other comparable NRAs (McAuslane et al., 2018). Furthermore, this could enable the application of an appropriate framework for benefit-risk assessment to enhance consistency in the clinical assessment of medicines (Leong et al., 2015) as well as incorporating the principles of GRevP in routine regulatory undertakings (WHO, 2014).

### Approval Times

As stated by Leng et al. (2015), “The MCC had been under considerable pressure to increase the rate of medicines registration and was accused of delaying patients’ access to affordable and essential medicines.” The outcomes of an investigation into delayed timelines for registration of medicines, initiated in 2006 by the Minister of Health, noted a lack of skilled human resources, poor infrastructure and inefficient regulatory processes as the major barriers affecting timely patient access to medicines (Green-Thompson, 2008). This demonstrated that the MCC, while in existence, neither achieved the target timelines set for the eligible applications of essential medicines that were assigned “fast track” status nor met the targets in 2015, 2016, and 2017 for the key milestones within the regulatory review process. Furthermore, the MCC made use of a manual system to track applications for market authorisation, but it is hoped that the imminent implementation of an electronic document management system by SAHPRA will promote systematic and formal communication regarding timelines and milestones to both internal and external stakeholders.

The MCC did not set a target for overall approval time of NAS applications. In order for SAHPRA to measure and improve its regulatory performance it is recommended that targets for overall approval time and key review milestones need to be identified, codified into policy and guidelines, recorded, measured and monitored (Keyter et al., 2018). Appropriate systems and resources now need to be put in place to ensure that regulatory performance metrics are analysed on a continuous basis through formal and routine monitoring resulting in measuring the key milestones in the regulatory review process including administrative and technical screening time, queuing time prior to review and clock stops measuring the time with sponsors. There is now the potential to improve regulatory review time through ongoing analysis of the performance metrics which may inform continuous improvement initiatives aimed at streamlining and prioritising the progression of the review process.

Review times may be improved as a result of the more flexible approach to committee structures that has been implemented by SAHPRA. Committee structures within SAHPRA have been revised to allow for more frequent *ad hoc* consultation with scientific committees, limited to applications for market authorisation requiring expert review and recommendation, as opposed to routinely channelling assessment reports through the committees for recommendation at 6-weekly intervals. Nevertheless, operationalisation of the current system may not produce satisfactory outcomes and therefore a more fundamental review of the entire agency could still be proved to be of value.

### Good Review Practices

The implementation of GRevP provides a mechanism for NRAs to enhance regulatory performance (World Health Organization [WHO], 2015a) and previous studies have demonstrated that regulatory performance indicators such as overall approval timelines can be enhanced by instituting quality management systems and GRevP into the regulatory review process (Cone and McAuslane, 2006). GRevP are a fundamental part of overall Good Regulatory Practice (GRP) with a focus on medical product review (World Health Organization [WHO], 2015b). GRevP are defined by the WHO as “documented best practices for any aspect related to the process, format, content and management of a medical product review” (World Health Organization [WHO], 2015b). The application of GRevP provides a platform for NRAs to “achieve timeliness, predictability, consistency, transparency, clarity, efficiency and high quality in both the content and management of reviews”; with a view to achieve successful review outcomes (World Health Organization [WHO], 2015b). Many NRAs have implemented systems to ensure the consistent application of GRevP and continue to work toward the evaluation and improvement of such systems.

The five regulatory authorities in this study implemented the majority of the essential elements of GRevP. The MCC did not have a dedicated quality department, however, there are plans to include dedicated quality personnel within the newly established SAHPRA. While key quality measures had been established and were evident in the work performed by the MCC, the need to formalise the quality management system, including the internal quality policy, GRevP systems, standard operating procedures and harmonised assessment templates must be prioritised in order to enhance SAHPRA operations. The establishment of a codified quality management system within SAHPRA should be supported by formally introduced continuous improvement measures such as internal and external quality audits that are routinely and formally implemented and the initiation of an electronic document management system.

The MCC had always recognised the importance of transparency and communication with stakeholders and as SAHPRA moves forward it is hoped that many of the measures that contribute toward transparency and communication will be formally and routinely implemented in an effort to enhance the consistency, timeliness and predictability of the review process. The imminent application of an electronic document management system will allow for improved transparency as sponsors will be able to track the progress of applications and overall approval times and the monitoring and measurement of key milestones in the review process will be readily available. However, whilst it is generally agreed that there are several aspects to review practices that are considered important, it is recognised that the “summary basis of approval” has far greater impact with respect to the regulatory process transparency than other relevant aspects (Vawda and Gray, 2017).

The MCC implemented a guideline in 2007 for the evaluation of benefit-risk assessment of medicines and prepares a summary basis of approval for each medicine evaluated; both of which are key steps in the regulatory review process. The clinical assessment of NASs was conducted by external experts who prepared assessment reports that were peer reviewed within the clinical committee structure. Without a standardised template for the clinical assessment report, informing regulatory decisions concerning the registration of a NAS relied heavily on the experience and expertise of such reviewers. SAHPRA may consider improving the current framework by building quality into the process and standardising the template used for benefit-risk assessment. SAHPRA may also consider implementing the Universal Methodology for Benefit-Risk Assessment (UMBRA) framework which has been assessed and applied by several mature NRAs (Walker et al., 2013) as well as NRAs in the emerging markets (Mashaki Ceyhan et al., 2018). This structured approach will promote improved consistency and predictability in the benefit-risk assessment of medicines as the use of the UMBRA framework “assists decision makers with clearly defining the decision, agreeing the requisite properties of the treatments being considered, assessing the trade-offs among these properties and making defensible and transparent decisions regarding the registration of the medicine” (Levitan et al., 2014).

Publication of the summary basis of approval is a norm for many mature NRAs globally and is a tool that can be used by regulatory authorities to build confidence in the review process in order to provide assurance regarding safety provisions (McAuslane et al., 2009). It is recommended that SAHPRA consider publishing the summary basis for approval, which were not previously made available in the public domain by the MCC. However, it is recognised that in order to achieve this outcome a change in legislation is required.

The data collected for the purpose of this study has allowed for a valuable comparison of regulatory authorities with similar regulatory mandates, similar size and similar resources characteristics. A number of recommendations have been provided with a view to inform areas of improvement that may be prioritised to underpin the success of SAHPRA as it moves toward goals of regulatory reform and enhanced regulatory performance.

### Recommendations

The comparison of the registration process previously applied by the MCC with those of similar medium-size regulatory authorities such as the TGA, Health Canada, the HAS, and Swissmedic has highlighted key areas for change and development. The following recommendations may be considered by SAHPRA in order to improve on the MCC regulatory review process.

• Defining target timelines for the key milestones in the regulatory review process and overall approval time and ensuring the formal and routine monitoring and measurement of such metrics.
• Formally implementing and maintaining GRevP in order to build quality into the review process, resulting in consistent, predictable, transparent and a timely regulatory review.
• Applying the universal methodology for benefit-risk assessment to enhance consistency in the clinical review of medicines and promote defensible and transparent decision making.
• Implementing facilitated regulatory pathways and applying a risk-based approach to the regulatory review process in order to conserve limited resources and avoid duplication of regulatory efforts.
• Establishing committee structures within the South African NRA should allow for *ad hoc* consultation limited to applications for market authorisation requiring expert review and recommendation.
• Enhancing transparency and communication through the development of summaries for the basis of approval that may be made available in the public domain.

## Data Availability

All datasets generated for this study are included in the manuscript and/or the supplementary files.

## Author Contributions

AK designed the study, collated and analysed the data, prepared the results, and wrote the manuscript. SW and SS designed the study, reviewed the results, and reviewed the manuscript. SB facilitated the data collection and reviewed the manuscript.

## Conflict of Interest Statement

AK was employed by the South African Health Products Regulatory Authority (SAHPRA), under the auspices of the South African National Department of Health. SB was appointed as a member of SAHPRA Board and is the Chair of SAHPRA Technical Operations and Regulatory Strategy (TORS) Committee. The remaining authors declare that the research was conducted in the absence of any commercial or financial relationships that could be construed as a potential conflict of interest.

## Abbreviations

CMC: chemistry, manufacturing and control (CMC)
CPP: certificate of pharmaceutical product
CTD: Common Technical Document (CTD)
GMP: good manufacturing practice
GRevPs: good review practices
HSA: (Singapore’s) Health Sciences Authority
MCC: Medicines Control Council
MLE: major line extension
NASs: new active substances
NRA: national regulatory authority
PIC/S: Pharmaceutical Inspection Co-operation Scheme
SAHPRA: South African Health Products Authority
TGA: (Australia’s) Therapeutic Goods Administration
